# Metal coordination polymer nanoparticles for cancer therapy

**DOI:** 10.1042/EBC20253012

**Published:** 2025-04-10

**Authors:** Zhengzheng Zhang, Isra Rana, Jutaek Nam

**Affiliations:** College of Pharmacy, Chonnam National University, Gwanju 61186, South Korea

**Keywords:** cancer immunotherapy, chemodynamic therapy, drug delivery, metal coordination polymer nanoparticle, metal ions, regulated cell death

## Abstract

Metal ions are essential elements in biological processes and immune homeostasis. They can regulate cancer cell death through multiple distinct molecular pathways and stimulate immune cells implicated in antitumor immune responses, suggesting opportunities to design novel metal ion-based cancer therapies. However, their small size and high charge density result in poor target cell uptake, uncontrolled biodistribution, and rapid clearance from the body, reducing therapeutic efficacy and increasing potential off-target toxicity. Metal coordination polymer nanoparticles (MCP NPs) are nanoscale polymer networks composed of metal ions and organic ligands linked via noncovalent coordination interactions. MCP NPs offer a promising nanoplatform for reshaping metal ions into more drug-like formulations, improving their *in vivo* pharmacological performance and therapeutic index for cancer therapy applications. This review provides a comprehensive overview of the inherent biological functions of metal ions in cancer therapy, showcasing examples of MCP NP systems designed for preclinical cancer therapy applications where drug delivery principles play a critical role in enhancing therapeutic outcomes. MCP NPs offer versatile metal ion engineering approaches using selected metal ions, various organic ligands, and functional payloads, enabling on-demand nano-drug designs that can significantly improve therapeutic efficacy and reduce side effects for effective cancer therapy.

## Introduction

Many metal ions are essential trace elements that play crucial roles in various fundamental biological processes, functioning as essential nutrients or cell/protein-specific regulators [1–3]. They are indispensable for modulating systemic immune responses and maintaining homeostasis [4–7]. Additionally, some metal ions have been developed into anticancer agents due to their inherent cytotoxicity [8,9]. This suggests potential opportunities for designing metal ion-based cancer therapies that not only directly kill cancer cells but also stimulate anticancer immunity. However, metal ions typically exhibit unfavorable *in vivo* biodistribution and poor target cell uptake due to their small size and high charge density, necessitating formulations that can enhance their pharmacological performance and therapeutic index for cancer therapy applications.

**Figure 1:**
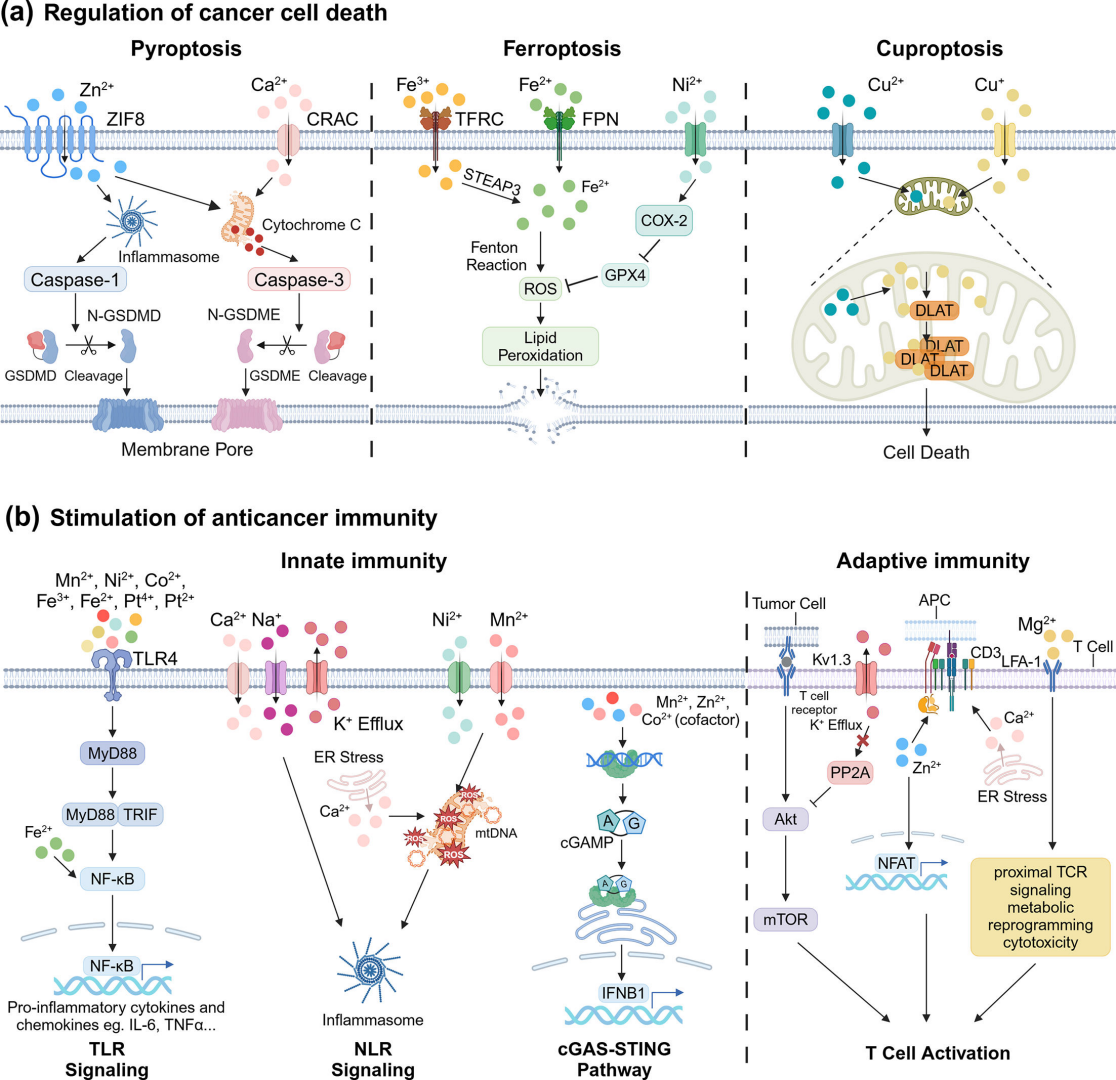
Roles of metal ions in cancer therapy. (**a**) Regulation of cancer cell death by various metal ions. (**b**) Stimulation of innate and adaptive immune responses by metal ions. The figure was created with BioRender.com.

Metal coordination polymer nanoparticles (MCP NPs) are nanoscale polymer networks composed of metal ions and organic ligands linked via noncovalent coordination interactions [10–12]. MCP NPs offer a promising nanoplatform that can deliver high dose of metal ions in a more effective manner with a favorable modulation of their physicochemical properties. This review focuses on the intrinsic biological activities of metal ions in regulating cancer cell death and stimulating anticancer immune responses, elucidating their mechanisms of action and potential applications in cancer therapy (Figure 1 and Table 1). We also discuss preclinical studies that highlight crucial design principles for engineering metal ions into MCP NPs for effective cancer therapy and provide the perspectives of MCP NPs for metal ion-based cancer treatment.

**Table 1:** Mechanisms and potential applications of bioactive metal ions for cancer therapy.

Metal ion	Mechanism	Outcome	Potential application	Reference
Zn^2+^	Activate caspase-1/GSDMD-dependent canonical pathway and caspase-3/GSDME-dependent alternative pathway Increase ROS level and stimulate autophagy pathway Activate the cGAS-STING pathway by binding to cGAS and stabilizing the cGAS-DNA complex Enhance Lck autophosphorylation via CD4/CD8 interactions and promote Th1 cytokine production	Pyroptosis Induce innate immunity Induce adaptive immunity	RCD inducer Immunotherapy candidate	[7,13–17]
Ca^2+^	Activate caspase-3 pathway Induce the dysfunction of mitochondria to generate ROS Activate NLRP3 inflammasome Promote CD8^+^ T cell activation	Pyroptosis Induce innate immunity Induce adaptive immunity	RCD inducer Immunotherapy candidate	[18–21]
Fe^2+ / 3+^	Amplify PLOOHs and increase ROS level Activate TLR4	Ferroptosis Antitumor immune response	RCD inducer Immunotherapy candidate	[22–24]
Pt^2+ / 4+^	Increase the expression of ferritin and the concentration of iron Stimulate TLR-dependent immune activation	Ferroptosis ICD	RCD inducer Immunotherapy candidate	[25,26]
Cu^2+^	Disrupt mitochondrial metabolism and bind to lipoylated proteins to trigger DLAT oligomerization, leading to cell death	Cuproptosis	RCD inducer	[27]
Mn^2+^	Accumulate ROS and release mitochondrial DNA to activate NLRP3 inflammasome Activate cGAS-STING pathway	Induce innate immunity	Immunotherapy candidate	[15,28]
Na^+^	Activate NLRP3 inflammasome	Induce innate immunity	Immunotherapy candidate	[4]
K^+^	Activate NLRP3 inflammasome Regulate CD8^+^ T cell function	Induce innate immunity Induce adaptive immunity	Immunotherapy candidate	[29,30]
Ni^2+^	Down-regulate GPX4 to increase ROS level Activate TLR4 and promote the release of inflammatory cytokines and chemokines Activate NLRP3 inflammasome	Ferroptosis Antitumor immune response Induce innate immunity	RCD inducer Immunotherapy candidate	[23,28,31]
Co^2+^	Activate TLR4 and increase the secretion of inflammatory cytokines Activate cGAS-STING pathway	Induce innate immunity	Immunotherapy candidate	[8,32]
Mg^2+^	Promote extravasation of T cells to stimulate TCR signaling	Induce adaptive immunity	Immunotherapy candidate	[33,34]

GSDM, gasdermin. Lck, leukocyte-specific protein tyrosine kinase. PLOOHs, phospholipid hydroperoxides. GPX4, glutathione peroxidase 4. DLAT, dihydrolipoamide S-acetyltransferase. ICD, immunogenic cell death. TCR, T cell receptor.

### Metal ions for regulating cell death

Metal ions play a pivotal role in regulating redox homeostasis, which is essential for cell survival [35–41]. Cancer cells often exhibit elevated levels of reactive oxygen species (ROS) due to their high metabolic activity, which can be amplified by metal ions, such as Cu²^+^, Zn²^+^, Mn²^+^, and Co²^+^. These ions skew the balance between oxidation and reduction reactions toward oxidative stress [42–46]. Such imbalance ultimately leads to cellular damage and cell death through apoptosis involving proteins such as p53 and MAPK kinase and subcellular impacts such as mitochondrial dysfunction, autophagy signaling, and oxidative DNA damage [13,18,45,47,48].

Moreover, metal ions can induce regulated cell death (RCD) controlled by specific genes and biomacromolecules, distinct from traditional cell death pathways [49]. Pyroptosis, ferroptosis, and cuproptosis are among the most studied and well-characterized RCDs. Pyroptosis is associated with the gasdermin (GSDM) protein family, such as GSDMD and GSDME, where the N-terminal fragment is released after cleavage by activated caspase-1 or caspase-3, generating membrane pores and ultimately leading to cell swelling, rupture, and death [49–51]. Zn^2+^ overload can cause pyroptosis via the caspase-1/GSDMD-dependent canonical pathway or the caspase-3/GSDME-dependent alternative pathway [14]. Ca^2+^ accumulation in mitochondria can release cytochrome C and subsequently activate caspase-3 for GSDME-dependent pyroptosis [19]. Ferroptosis is characterized by the accumulation of lipid peroxides in cell membranes, regulated by metabolic enzymes such as lipoxygenases and cytochrome P450 oxidoreductase involved in phospholipid peroxidation [52,53]. Fe^2+^ and Fe^3+^ can catalyze the metabolic enzymes to amplify phospholipid hydroperoxides, the hallmark of ferroptosis [22]. Ni^2+^ can up-regulate cyclooxygenase 2 and down-regulate glutathione peroxidase 4 to induce ferroptosis [23]. Cisplatin was reported to increase the expression of ferritin and transferrin receptor linked to iron-dependent ferroptosis [25]. Cuproptosis is associated with the dysregulation of mitochondrial respiration and metabolism [54]. Cu²^+^ plays a pivotal role in cuproptosis by binding to the lipoylated dihydrolipoamide S-acetyltransferase (DLAT), triggering DLAT oligomerization and proteotoxic stress that leads to cell death [27].

### Metal ions for stimulating anticancer immune cells

Numerous metal ions can engage various immune sensors and receptors, triggering downstream signaling pathways that promote the activation and maturation of immune cells [55–58]. Innate immunity, the first line of defense, provides rapid and nonspecific protection against foreign pathogens and immunogens [59,60]. Key players in the innate immune system include professional antigen-presenting cells (APCs) such as dendritic cells (DCs) and macrophages. These cells bridge the activation of T cells via immune synapse signaling, conferring robust and durable antigen-specific adaptive immune responses [58,60]. Particularly, CD8^+^ T cells are critical effectors in adaptive immunity that can directly target and eliminate cancer cells through cellular immune responses.

Some metal ions can stimulate the activation of APCs via pattern recognition receptors such as toll-like receptors (TLRs) [61], nucleotide-binding oligomerization domain-like receptors (NLRs) [28], and stimulator of interferon genes (STING) [62]. TLRs, located on plasma or endosomal membranes, trigger inflammatory cascades via MyD88/TRIF and NF-κB pathways [63]. Mn²^+^, Ni²^+^, Co²^+^, Pt²^+^/⁴^+^, and Fe²^+^/³^+^ have been reported to stimulate TLRs directly or indirectly [24,26,31,32,64]. NLR signaling promotes inflammasome formation and downstream production of pro-inflammatory cytokines [65]. Dysregulated cellular levels of Na^+^, K^+^, and Ca²^+^ can activate NLRs as damage-associated molecular patterns (DAMPs) associated with hyperosmotic stress [66]. Epithelial sodium channels and the phosphatidyl-inositol/Ca²^+^ pathway are implicated in NLRP3 inflammasome activation by Na^+^/K^+^ and Ca²^+^, respectively [4,29]. Mn^2+^ and Ni^2+^ can promote inflammasome activation by inducing mitochondrial damages and ROS accumulation [20,28]. For STING signaling, cyclic GMP-AMP synthase (cGAS) detects cytosolic double-stranded DNA and catalyzes the synthesis of cyclic GMP-AMP that stimulates STING to induce the production of type I interferons and pro-inflammatory cytokines [63,67]. Mn²^+^, Zn²^+^, and Co²^+^ have been reported to promote cGAS-STING pathway activation [8,15]. Zn²^+^ binds to cGAS and stabilizes the cGAS-DNA complex, while Mn²^+^ acts as a cofactor to sensitize and activate both cGAS and STING [6,16]. Additionally, these ions facilitate the translocation of the STING complex from the endoplasmic reticulum to Golgi compartments, essential for the signaling cascade [6].

Some metal ions can also directly impact T cells [68]. K^+^ is a crucial regulator of CD8^+^ T cell function and stemness; K^+^ efflux through the Kv1.3 channel enhances T cell effector functions [30,69,70]. Mg²^+^ sensitizes the leukocyte function-associated antigen-1 (LFA-1), promoting T cell trafficking and extravasation [33], and induces conformational changes in LFA-1, stimulating the T cell receptor signaling pathways [34]. Ca²^+^ promotes CD8^+^ T cell activation by triggering CD3 tyrosine phosphorylation and sensitizing T cells toward the major histocompatibility complex [21]. Zn^2+^ enhances leukocyte-specific protein tyrosine kinase autophosphorylation via CD4/CD8 interactions, supporting T cell activation and Th1 cytokine production (IFN-γ, IL-2) for effective cellular immunity [7,17].

### MCP NPs in cancer therapy

**Figure 2:**
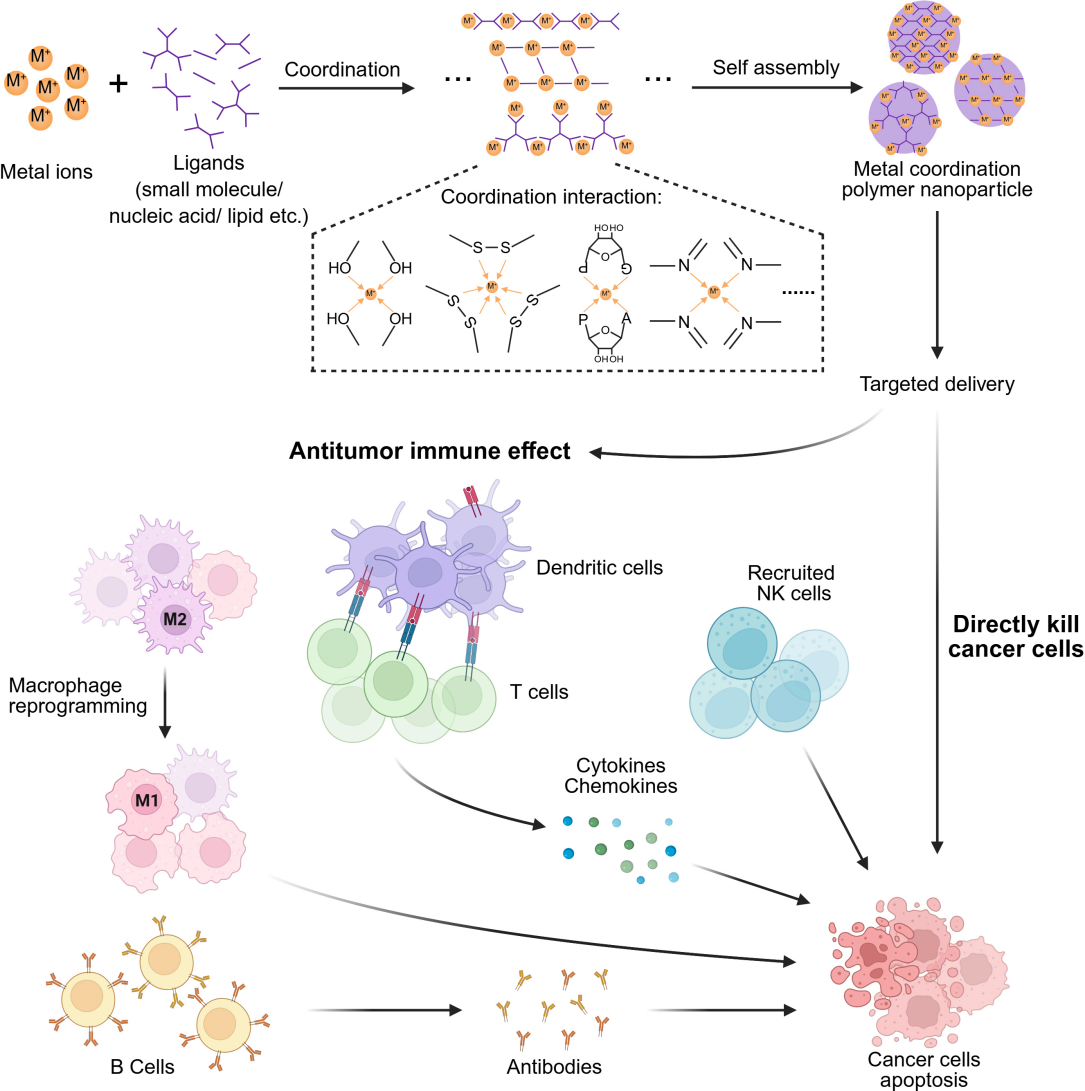
Application of metal coordination polymer nanoparticles (MCP NPs) in cancer therapy. MCP NPs can be developed using diverse metal ions and coordination ligands for distinct functional activities, enabling effective cancer therapy through direct cancer cell death and stimulation of antitumor immune responses. The figure was created with BioRender.com.

The intrinsic biological functions of metal ions suggest their potential as anticancer drugs. However, unfavorable pharmacological properties often necessitate high-dose administration, potentially causing side effects and toxicity due to physiological imbalances at nontargeted sites [71]. Various strategies have been explored to achieve controlled delivery and efficacy, such as using ionophores, antibody–metal conjugates, or metal ion-containing drug conjugates [3,72–74]. Recently, nanomedicine approaches have emerged to engineer sophisticated nanoplatforms for metal ions [3,75]. Notably, NP formulations can alter the physicochemical properties of metal ions, preferentially modulating their *in vivo* pharmacological profiles to improve therapeutic efficacy while reducing off-target toxicity. In particular, inorganic–organic hybrid MCP NPs are gaining increased attention as a promising metal ion delivery platform [76,77]. Compared with conventional metal NPs created by strong metallic bonds between reduced metal ions, MCP NPs can preserve the natural form of metal ions as they produce NPs with organic ligands via the noncovalent coordination interaction. Furthermore, MCPs offer versatile metal ion engineering approaches using various organic ligands that contain metal coordination groups, such as carboxyl, amino, heterocyclic oxygen, or nitrogen [78]. MCP NPs are prepared by simply mixing metal ions and organic ligands under mild conditions, eliminating the need for toxic reducing agents, high temperatures, organic solvents, and inert atmospheres typically required for the controlled synthesis of conventional metal NPs [79]. The selection of metal ions and ligands not only determines the composition and structure but also influences the biological functions of MCP NPs [80,81]. This attribute also distinguishes MCP NPs from conventional metal NPs that lack coordination ligands and thus pose limited functionality. In addition, the weak noncovalent intramolecular interaction of MCP NPs can be dissociated and cleared *in vivo*, potentially alleviating the toxicity issues associated with poorly degraded metal NPs due to strong metallic bonds. In the following sections, we explore various design aspects of MCP NP systems, focusing on their preclinical cancer therapy applications, including chemodynamic therapy (CDT), chemotherapy, phototherapy, and cancer vaccines. Drug delivery principles play a crucial role in optimizing their therapeutic outcomes (Figure 2 and Table 2).

**Table 2:** Examples of metal coordination polymer nanoparticle systems for cancer therapy applications

Applications	MCPs formulation	Properties and outcomes	Reference
Chemodynamic therapy	Fe^2+^/DPA/HA/β-lap	Generate ROS via Fenton reaction and trigger calcium overload and ferroptosis pathway	[82]
	Fe^3+^/CHC	Trigger acidosis to facilitate the Fe^3+^-mediated Fenton reaction, enhancing the CDT effect	[83]
	Mn^2+^/AQ4N / DA	Exacerbate tumor microenvironment hypoxia, and accelerate Mn^2+^-mediated Fenton-like activity, enhancing the effect of CDT	[84]
	Cu^2+^/IR783	Deplete the GSH in TME thus enhance the Cu^+^-mediated Fenton-like reaction, and the overload of Cu ions trigger tumor-specific cuproptosis-mediated cell death	[85]
	Fe^3+^/EGCG/Pt-OH	Elevate the intracellular H_2_O_2_ level and generate ROS via Fe^3+^-mediated Fenton reaction	[86]
	Cu^2+^/ DA/DOX	Amplify oxidative stress at the cellular level by generating •OH and depleting intracellular GSH	[87]
Chemotherapy	Pt(II)/ GA/ methoxy-PEG	Release CDDP in response to acidic pH and ROS, achieving prolonged circulation, enhanced tumor accumulation, and improved antitumor efficacy with reduced toxicity	[88]
	Cu^2+^/BSA/GA/DOX	Convert GSH to GSSG and generate ROS via Fenton-like reaction, enhancing CDT	[89]
	Fe^2+^/Sorafenib/DOX	Release DOX in response to elevated GSH levels in the TME, and the accumulated Fe^2+^ leads to ferroptosis	[90]
	Mn^2+^/MTX/PEG	Release MTX in response to the change in pH and allow tumor-targeted accumulation	[91]
	Fe^3+^/MTX	Promote antigen presentation, immune activation, T cell infiltration, and boost the therapeutic effect of immune checkpoint blockade therapy	[92]
	Fe^3+^/TA /DOX	Release DOX and Fe^3+^ in response to pH, and boost ICD of cancer cells through chemotherapy and ferroptosis	[93]
	Zn^2+^/2-MIM/TP5/MTX	Zn^2+^ disrupts glycolytic process and induces PD-L1 protein degradation via AMPK pathway; MTX induces ICD and dsDNA damage to activate cGAS-STING pathway	[94]
Phototherapy	Zn^2+^/ICG / DPA	Zn^2+^ coordinates with ICG, allowing NIR PTT, and carries therapeutic siRNA, combined with phototherapy, to enhance therapeutic efficacy	[95]
	Metal ions (Mn^2+^, Ca^2+^, and Co^2+^)/ICG/pHis-PEG	Provide a low-temperature PTT strategy	[96]
	Mn^2+^/PpIX	Produce ROS under light or ultrasound excitation for efficient killing of cancer cells	[97]
	Fe^3+^/HCA/DOX	Enable controlled release of DOX under low pH condition or NIR irradiation, enhancing antitumor efficiency	[98]
	Ru^2+^/Fe^2+^/MTX-SA/siRNA	Ru^2+^ allows PDT, generating ROS with 670 nm irradiation, and induces ICD; Fe^2+^ triggers ferroptosis	[99]
Cancer vaccine	Zn^2+^/2-MIM/CpG ODNs	Enhanced intracellular uptake of CpG by macrophages and increased cytokine secretion	[100]
	Fe^3+^/terephthalic acid /CpG	Deliver CpG and increase cytokine secretion by macrophages	[101]
	ZIF8/OVA/CpG ODNs	Deliver OVA and CpG into APCs and promote release in response to low pH, and stimulate humoral and cellular immune responses	[102]
	Mn^2+^/BPNS/CpG ODNs	Activate TLR9 and STING pathways to active DCs and enhance T cell activation	[103]
	Fe-MOF/OVA/CpG	Increase cytokines and OVA-specific CTL responses and induce immune memory response	[104]
	Eu^3+^/GMP/OVA/CpG	Enhance Th1 immune responses, increase the CD8^+^ T cells, and increase the infiltration of tumor-killing immunocytes	[105]
	MXF/DNA (CpG)	Enhance radiotherapy and induce ICD of cancer cells	[106]

DPA, dithiodipropionic acid. HA, hyaluronic acid. β-lap, β-lapachone. CHC, α-cyano-4-hydroxycinnamate. DA, dopamine. GSH, glutathione. EGCG, epigallocatechin-3-gallate. GA, gallic acid. CDDP, cisplatin. DOX, doxorubicin. MTX, methotrexate. TA, tannic acid. 2-MIM, 2-methylimidazole. TP5, thymopentin. ICG, indocyanine green. DPA, dipicolylamine. PpIX, protoporphyrin Ⅸ. HCA, hydrocaffeic acid. MTX-SA, mitoxantrone-succinic acid. 2-MIM , 2-methylimidazole. CpG ODNs, CpG oligodeoxynucleotides. ZIF-8, zeolitic imidazolate framework-8. OVA, ovalbumin. CTL, cytotoxic T lymphocyte. BPNS, black phosphorus nanosheet. Fe-MOF , Fe^3+^-aminoterephthalate metal–organic framework. MXF , metal 'X' frameworks. ICD, immunogenic cell death. TME, tumor microenvironment.

## Chemodynamic therapy (CDT)

CDT utilizes Fenton or Fenton-like reactions of catalytic metal ions to induce ROS-mediated cell death [107]. MCP NPs can harness metal ions for ROS production and can also be designed to incorporate functional ligands that leverage specific cues in the tumor microenvironment and cancer cells to increase target-specific activity. For instance, Pan et al. prepared MCP NPs through coordination of Fe^2+^ and 3,3′-dithiodipropionic acid, and then further deposited CaO_2_ NPs, β-lapachone (β-lap), and hyaluronic acid (HA) to obtain HCF@β-lap [82]. HCF@β-lap efficiently targeted CD44 over-expressing tumors with surface functionalized HA, disintegrating through cleavage of disulfide links by intracellular glutathione (GSH). Subsequently released CaO_2_ and β-lap amplified H_2_O_2_ production and generated O_2_ to alleviate tumor hypoxia, inducing severe oxidative damage together with the Fenton reaction of self-supplied Fe^2+^. Chi et al. constructed self-assembled NPs using Fe^3+^ and α-cyano-4-hydroxycinnamate (CHC) [83]. CHC promoted acidosis by inhibiting transmembrane monocarboxylate transporter 4, facilitating the Fe^3+^-mediated Fenton/Fenton-like reactions for enhanced CDT efficacy. Chang et al. designed MCP NPs using a hypoxia-activatable prodrug AQ4N, Mn^2+^, and dopamine [84]. The NPs utilized tumor hypoxia to control drug activity, while Fenton-like reaction of Mn^2+^ further improved therapeutic efficacy. Hu et al. developed MCP NPs comprising Cu^2+^ and sonosensitizer IR783 [85]. IR783 enabled NIR fluorescence imaging and sonodynamic therapy (SDT), while Cu^2+^ depleted GSH and induced Cu^+^-mediated Fenton-like reaction and cuproptosis in tumors, leading to effective cancer therapy. Ren et al. integrated epigallocatechin-3-gallate and 5-hydroxydopamine-modified platinum prodrug (Pt-OH) into MCP NPs via coordination between Fe^3+^ and polyphenols [86]. The acidic endosomal environment in cancer cells triggered the release and conversion of Pt-OH into cisplatin, elevating intracellular H_2_O_2_ level through cascade reactions and amplifying therapeutic effect of Fe^3+^-catalyzed Fenton reaction. Xiong et al. constructed MCP NPs using Cu^2+^, dopamine, and redox-responsive hydroxyethyl starch prodrugs (HES-SS-DOX) [87]. Inclusion of Cu^2+^ enhanced NIR absorption, enabling thermally boosted chemo-/CDT, where the reduction in Cu^2+^ and disulfide bonds by GSH amplified oxidative stress within cancer cells.

## Chemotherapy

Chemotherapy is a standard cancer treatment, yet traditional small molecule-based drugs exhibit a narrow therapeutic window due to uncontrolled biodistribution and toxicity [108,109]. MCP NPs can enhance the targeted delivery of chemotherapeutic drugs, thereby broadening their therapeutic window. For example, Xiang et al. developed MCP NPs via the coordination of the Pt-containing chemotherapeutic drug cisplatin (CDDP) and methoxy-PEG terminated with gallic acid [88]. The resulting NPs exhibited prolonged blood circulation, efficient tumor accumulation, and prompt release of CDDP in response to elevated acidity and ROS levels in tumors, resulting in improved antitumor efficacy and reduced toxicity compared with free CDDP.

MCP NPs can also be engineered with metal ions possessing inherent anticancer activity and/or bioimaging capability. Zhang et al. utilized bovine serum albumin for *in situ* coordination of gallic acid and Cu²^+^, along with the adsorption of doxorubicin (DOX), for combined chemo-CDT [89]. The MCP NPs released Cu²^+^ and DOX under acidic condition, leading to efficient tumor inhibition through Cu²^+^-mediated Fenton-like reaction paired with DOX-induced chemotherapy. Xie et al. self-assembled Fe²^+^, sorafenib, and DOX into MCP NPs, in which Fe²^+^ acts as a ferroptosis inducer, sorafenib as a ROS resistance inhibitor, and DOX as a chemotherapeutic agent [90]. The NPs accumulated efficiently in tumors and released drugs in response to elevated GSH levels, inducing strong anticancer effects via synergistic ferroptosis and chemotherapy. Wu et al. developed MCP NPs using methotrexate (MTX) and Mn²^+^, achieving simultaneous MTX-mediated chemotherapy and Mn²^+^-induced magnetic resonance imaging (MRI) for theranostic cancer treatment [91].

Certain chemotherapeutic drugs can induce immunogenic cell death (ICD) that triggers anticancer immune responses [110]. Yu et al. demonstrated that MTX-Fe³^+^ coordination NPs can induce ICD characterized by the release or exposure of DAMPs, such as HMGB1, ATP, and CRT. This promoted the activation and tumor infiltration of CD4^+^ and CD8^+^ T cells, enhancing the therapeutic response to immune checkpoint blockade therapy [92]. Similarly, Xu et al. showed that DOX-loaded Fe^3+^–tannic acid coordination NPs can boost ICD of cancer cells [93]. Zhang et al. engineered zeolitic imidazolate framework-8 (ZIF-8) NPs, a well-known coordination system between Zn^2+^ and 2-methylimidazole, to encapsulate MTX and thymopentin (TP5) [94]. It was demonstrated that Zn^2+^ could disrupt the glycolytic process, depriving cancer cells of energy and inducing PD-L1 protein degradation via the AMPK pathway. MTX activated the cGAS-STING pathway by inducing ICD and dsDNA damage, while TP5 promoted the proliferation and differentiation of DCs and T cells. Consequently, the engineered NPs efficiently sensitized cancer cells to antitumor immune responses, achieving potent chemo-immunotherapy.

## Phototherapy

Phototherapy is gaining traction as a minimally invasive and highly selective treatment for solid tumors, allowing precise spatial control over therapeutic interventions. Two primary forms of phototherapy are photothermal therapy (PTT) and photodynamic therapy (PDT). These approaches typically employ exogenous photosensitizers that can absorb incident light and subsequently undergo energy transfer processes to generate localized heating (PTT) or ROS (PDT) for killing cancer cells [111,112]. Despite the promise, organic dyes used as photosensitizers face challenges such as low absorption cross-sections, photobleaching, and poor tumor specificity [113]. MCP NP formulations can enhance the optical properties and tumor-specific accumulation of organic dye photosensitizers.

Indocyanine green (ICG) is an FDA-approved near-infrared (NIR) dye extensively explored for both PTT and PDT applications [114,115]. Chu et al. developed MCP NPs based on Zn²^+^ and dipicolylamine, incorporating ICG, siRNA, and the tumor-targeting RGD peptide [95]. ICG facilitated NIR PTT, while siRNA down-regulated PTT-resistant survivin and HSP70, collaboratively suppressing RGD-targeting tumors. Yang et al. demonstrated that ICG could form MCP NPs with several metal ions, including Mn²^+^, Ca²^+^, and Co²^+^ [96]. Interestingly, the structure displayed 1D nanofiber-like morphologies in aqueous environments due to metal coordination and hydrogen bonding with water molecules. This configuration enabled low-temperature PTT in combination with co-delivered gambogic acid that inhibits thermoresistant HSP90. Porphyrin derivatives are also widely investigated organic photosensitizers [116,117]. Geng et al. prepared MCP NPs using Mn²^+^ and protoporphyrin IX (PpIX), where PpIX functions as a sensitizer for both PDT and SDT, enabling efficient cancer cell eradication through combined treatment [97].

Intriguingly, metal coordination interactions can impart novel optical properties suitable for phototherapy application. Li et al. reported that Fe^3+^ and hydrocaffeic acid MCP NPs exhibit NIR activity via ligand-to-metal charge transfer between phenolic oxygen and Fe^3+^ [98]. The MCP NPs released loaded DOX under acidic and high-temperature conditions due to the pH/heat-sensitive nature of coordination interactions, leading to robust anticancer efficacy through combined chemo-PTT therapy. An alternative approach involves metal-to-ligand charge-transfer complexes, such as ruthenium(II) polypyridyl derivatives, which exhibit strong optoelectronic properties [118–120]. Li et al. constructed MCP NPs by coordinating carboxylated ruthenium(II) polypyridyl derivatives and mitoxantrone–succinic acid with Fe²^+^, demonstrating effective combination therapy utilizing PDT, ROS-boosting siRNA, and Fe²^+^-catalyzed ferroptosis [99].

## Cancer vaccine

Cancer vaccines aim to license APCs using cancer antigens and immuno-adjuvants to prime T cells. MCP NPs can enhance targeted delivery to APCs, thereby improving vaccine efficacy and safety [121]. Here, we introduce the design of MCP NP vaccine systems using CpG oligonucleotide as a prominent example. CpG is a TLR-9 agonist and one of the most studied immuno-adjuvants in cancer vaccine applications [122–125]. Zhang et al. utilized positively charged, porous ZIF-8 NPs for the complexation and delivery of negatively charged CpG, demonstrating enhanced uptake of CpG and increased cytokine secretion by macrophages [100]. Yang et al. reported the similar results with MCP NPs constructed using Fe^3+^, terephthalic acid, and CpG, with Fe^3+^ also providing T2-weighted MRI capabilities [101]. Zhang et al. further demonstrated that ZIF-8 NPs could efficiently co-deliver cancer antigen ovalbumin (OVA) and CpG into APCs [102]. The NPs released their payloads in the low pH of endo-lysosomes, stimulating potent cytokine secretion and subsequent activation of CD4^+^ and CD8^+^ T cells. Similarly, Yang constructed MCP NPs using Fe^3+^ and aminoterephthalate [104], and Duan et al. used Eu^3+^ and guanine monophosphate for co-delivery of OVA and CpG [105]. Ling et al. designed Mn^2+^/CpG-decorated black phosphorus nanosheet (BPNS) through Mn^2+^ coordination to both BPNS and CpG [103]. CpG and Mn²^+^-induced immune stimulation, combined with antigen release by BPNS-mediated PTT and Mn²^+^-induced CDT, effectively activated APCs, leading to strong antitumor T cell responses.

Recently, direct supramolecular assembly of DNA/RNA and metal ions has garnered attention due to the rich coordination sites provided by oligonucleotides [126,127]. For instance, Yang et al. reported a series of nanoscale metal ‘X’ frameworks (MXFs) composed of various metal ions (e.g. Zn²^+^, Hf⁴^+^, and Ca²^+^) and DNA sequences [106]. The MXFs act as carrier-free nano-drugs when metal ions and DNAs both exhibit distinct functional activities, as demonstrated with Hf-CpG MXF; Hf concentrates X-ray radiation to enhance radiotherapy, while CpG serves as an immuno-adjuvant, enabling combined radio-immune therapy. Nanoscale metal coordination systems have also proven effective for the packing, protection, and delivery of various functional RNAs, maintaining their integrity and biological function [128], suggesting MCP NPs as promising platforms to overcome stability and delivery challenges associated with RNA pharmaceutics [129–131].

### Conclusions and perspectives

The inherent biological functions of metal ions present opportunities for designing novel metal ion-based cancer therapies. MCPs offer a promising platform technology for transforming metal ions into more drug-like formulations by favorably modulating their physicochemical properties to enhance pharmacological performance. In addition, the flexible design of MCPs provides several advantages as demonstrated by the preclinical studies: (1) MCP NPs can be synthesized using a diverse array of metal ions and organic ligands tailored to desired biological activities for specific applications; (2) the physicochemical properties, such as size, shape, porosity, and functionality can be finely tuned for optimal *in vivo* behavior; (3) different types of drugs, from small molecules to large biomacromolecules, can be co-incorporated into the coordination polymer network through noncovalent interactions such as coordination bonds, electrostatic interactions, hydrogen bonding, hydrophobic interactions, and π-π interactions; and (4) these noncovalent interactions can be selectively disrupted by various endogenous and exogenous stimuli, such as pH, GSH, ROS, and heat, providing controlled drug release and activity, thus increasing therapeutic efficiency and reducing side effects. Importantly, MCP NPs can serve as a carrier-free nano-drug platform when both metal ions and organic ligands function as active drug ingredients, potentially mitigating the safety issues associated with artificial nanocarriers used for drug delivery. Therefore, MCP NPs represent versatile drug delivery platforms that can improve the safety and efficacy of metal ion-based cancer therapies. Nonetheless, there remain several challenges to address for the widespread biomedical applications of MCP NPs. The physicochemical properties and surface functionalization of MCP NPs can regulate their interaction with biological systems and thus determine their *in vivo* performance. Therefore, it is imperative to establish a robust, scalable, and consistent manufacturing protocol for reliable evaluation and optimization of MCP NPs. The dose, frequency, and administration routes should also be optimized to maximize therapeutic efficacy while minimizing systemic side effects. Preclinical studies have demonstrated the benefits of MCP NPs to promote tumor-targeted drug delivery with improved biodistribution profiles. However, mononuclear phagocyte systems (MPS), particularly in the liver and spleen, can also massively accumulate them through nonspecific phagocytosis [87,132,133]. Although *in vivo* dissociation of MCP NPs and subsequent excretion of metal ions and other organic components via renal clearance have been reported on some occasions [134,135], their nonspecific distribution in MPS organs poses potential toxicity issues. Despite trace metal ions such as Cu²^+^, Fe²^+^/³^+^, and Mn^2+^ being generally considered nontoxic, their NP formulation can change intrinsic functional properties that are potentially associated with cytotoxicity. The definitive factors for the toxicology of NPs have not been established yet, requiring case-by-case evaluation of individual NP systems with standardized protocols. Given that the long-term toxicity issue is a major obstacle to the clinical translation of NP drugs, both therapeutic efficacy and long-term toxicity should be carefully investigated for individual MCP NPs in relation to their *in vivo* stability, biodistribution, and biodegradation profiles.

**Summary**

- Metal ions can regulate cancer cell death directly or indirectly via distinct signaling pathways and/or by stimulating antitumor immune cells.
- Metal coordination polymer nanoparticles (MCP NPs) offer a promising nanoplatform for transforming metal ions into more drug-like formulations by modulating their physicochemical properties.
- MCP NPs enable versatile nano-drug designs using a diverse array of metal ions, organic ligands, and functional payloads, significantly improving therapeutic efficacy and reducing side effects for effective cancer therapy.
- The manufacturing protocols and treatment regimens should be optimized with a thorough evaluation of *in vivo* therapeutic efficacy and long-term toxicity for further development of MCP NPs.

## Abbreviations

APCs: antigen presenting cells
BPNS: black phosphorus nanosheet
CDDP: cisplatin
CDT: chemodynamic therapy
CHC: α-cyano-4-hydroxycinnamate
DAMPs: damage-associated molecular patterns
DCs: dendritic cells
DLAT: dihydrolipoamide S-acetyltransferase
GSDM: gasdermin
GSH: glutathione
HA: hyaluronic acid
ICD: immunogenic cell death
ICG: indocyanine green
LFA-1: leukocyte function-associated antigen 1
MCP NPs: metal co-ordination polymer nanoparticles
MRI: magnetic resonance imaging
MTX: methotrexate
MXF: metal 'X' frameworks
NIR: near-infrared
NLRs: nucleotide-binding oligomerization domain-like receptors
OVA: ovalbumin
PDT: photodynamic therapy
PTT: photothermal therapy
PpIX: protoporphyrin Ⅸ
Pt-OH: 5-hydroxydopamine-modified platinum prodrug
RCD: regulated cell death
ROS: reactive oxygen species
SDT: sonodynamic therapy
STING: stimulator of interferon genes
TLRs: toll-like receptors
TME: tumor microenvironment
TP5: thymopentin
ZIF-8: zeolitic imidazolate framework-8
cGAS: cyclic GMP-AMP synthase
β-lap: β-lapachone
